# Rhinovirus is an important pathogen in upper and lower respiratory tract infections in Mexican children

**DOI:** 10.1186/s12985-015-0262-z

**Published:** 2015-02-26

**Authors:** Fernando E Aponte, Blanca Taboada, Marco A Espinoza, María A Arias-Ortiz, Jesús Monge-Martínez, Rubén Rodríguez-Vázquez, Fidel Díaz-Hernández, Fernando Zárate-Vidal, Rosa María Wong-Chew, Verónica Firo-Reyes, Carlos N del Río-Almendárez, Jesús Gaitán-Meza, Alberto Villaseñor-Sierra, Gerardo Martínez-Aguilar, Maricela García-Borjas, Daniel E Noyola, Luis F Pérez-Gónzalez, Susana López, José I Santos-Preciado, Carlos F Arias

**Affiliations:** Instituto de Biotecnología, Universidad Nacional Autónoma de México, Av.Universidad 2001, Colonia Chamilpa, Cuernavaca, Morelos 62210 Mexico; Colegio de Pediatría del Estado de Veracruz, Veracruz, Mexico; Facultad de Medicina, Universidad Nacional Autónoma de México, México, D F Mexico; Hospital General de México, México, D F Mexico; Hospital Pediátirco de Coyoacán, México, D F Mexico; Nuevo Hospital Civil de Guadalajara “Dr. Juan I. Menchaca”, Guadalajara, Jalisco Mexico; Centro de Investigación Biomédica de Occidente, IMSS, Guadalajara, Jalisco Mexico; Hospital General de Durango, Durango, Mexico; Universidad Autónoma de San Luis Potosí, San Luis Potosí, Mexico; Hospital Central “Dr. Ignacio Morones Prieto”, San Luis Potosí, Mexico

**Keywords:** Rhinovirus, Respiratory virus infections, Genetic diversity, Children

## Abstract

**Background:**

Most of the studies characterizing the incidence of rhinovirus (RV) have been carried out in hospitalized children and in developed countries. In those studies, RV-C has been associated with more severe respiratory tract infections than RV species A and B. In this study we determined the frequency and diversity of RV strains associated with upper and lower respiratory tract infections (URTI, LRTI) in Mexico, and describe the clinical characteristics of the illness associated with different RV species.

**Methods:**

A prospective surveillance of 526 and 250 children with URTI and LRTI was carried out. Respiratory samples were analyzed by RT-PCR for viruses. The 5′ untranslated region of the RV genome was amplified and sequenced.

**Results:**

In the case of URTI, 17.5% were positive for RV, while this virus was found in 24.8% of LRTI. The RV species was determined in 73 children with URTI: 61.6% were RV-A, 37% RV-C and, 1.4% RV-B; and in 43 children with LRTI: 51.2% were RV-A, 41.8% RV-C, and 7% RV-B. No significant differences in clinical characteristics were found in patients with RV-A or RV-C infections. A high genetic diversity of RV strains was found in both URTI and LRTI.

**Conclusions:**

Both RV-A and RV-C species were frequently found in hospitalized as well as in outpatient children. This study underlines the high prevalence and genetic diversity of RV strains in Mexico and the potential severity of disease associated with RV-A and RV-C infections.

## Introduction

Pneumonia continues to be a major killer of young children in developing countries and elderly people in developed countries. Despite recent advances, further studies are needed to examine regional variation in its etiology, particularly in developing countries, where most of the deaths from serious respiratory diseases occur [1,2].

Rhinovirus (RV) is the most frequent cause of acute respiratory illness worldwide [3-5]. This virus has been typically associated with upper respiratory tract infections (URTI); however, with the development of molecular methods RV has been found to be also commonly associated with lower respiratory tract infections (LRTI). These findings are changing the long held view of RV as a minor pathogen, as it is now being involved in a wide variety of respiratory illnesses, ranging from mild common colds and asthma exacerbation to serious lower respiratory tract disease [3-5].

RVs are antigenically quite diverse; historically, these viruses were classified into 99 serotypes through neutralization assays. These different RV serotypes were later classified genotypically into two species, RV-A (74 serotypes/genotypes) and RV-B (25 serotypes/genotypes) [6]. More recently, based only on genomic sequence information, a third virus species, RV-C, was described [6-9]. The current genotypification of these viruses is based on the nucleotide sequence encoding the VP1 and VP4/VP2 proteins [3]. Nevertheless, a variable region in the 5′-untranslated region (UTR) of the viral genome can also accurately distinguish among virus species [10]. Since its description in 2006, RV-C was suggested to be associated with more severe respiratory illness as compared to RV-A and RV-B, as well as with more frequent asthma exacerbations [11-16]. However, other studies have found no difference in illness severity among RV species [17-20], thus, more information is needed to clarify this issue.

Most studies have described the presence of RV genotypes in hospitalized patients with severe respiratory illness, and only a few studies have described the prevalence of virus genotypes in URTIs. In this work, we carried out a prospective multicenter study of two children populations having either URTI or LRTI.

## Results

### RV detection

**Figure 1:**
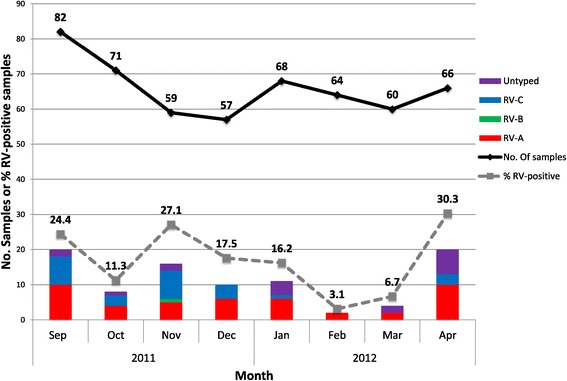
**Seasonal distribution of different RV species in children with upper respiratory tract infections.** The number of RV-A, RV-B, RV-C, and untyped RV-positive samples for each month are shown. The number of samples analyzed and the percentage of RV-positive samples per month are indicated.

Nasopharyngeal samples from 526 pediatric patients with URTI attending the private consult were tested for the presence of RV and other respiratory viruses. Ninety-two (17.5%) children (47 males and 45 females, age range 0 to 175 months) were RV positive. The species of 73 (80.2%) of these RVs was determined by sequencing the 5′-UTR region of the viral genome; 45 (61.6%) were RV-A, 27 (37%) RV-C, and 1 (1.4%) was RV-B (Table 1). The incidence of RV infection was highest in September and November 2011 as well as in April 2012, when it accounted for 24.4%, 27.1% and 30.3%, respectively, of the total samples collected (Figure 1).

**Table 1:** **Frequency of RV infections in children with upper and lower respiratory tract infections**

	**RV-positive**			**RV-A** ^**a**^			**RV-B** ^**a**^	**RV-C** ^**a**^			**p-value**
	**Total (%)** ^**b**^	**Single infection (%)**	**Co-infection (%)**	**Total (%)**	**Single infection (%)**	**Co-infection (%)**	**Total (%)**	**Total (%)**	**Single infection (%)**	**Co-infection (%)**	**RV-A vs. RV-C**
**URTI**	92 (17.5)	64 (12.2)	28 (5.3)	45 (8.6)	31 (5.9)	14 (2.7)	1 (0.2)	27 (5.1)	22 (4.2)	5 (0.9)	**0.027** ^**c**^
**Total samples (n=526)**											
**Age (months)**											
≤12 (n=126)	26 (20.6)	18 (14.3)	8 (6.3)	12 (9.5)	9 (7.1)	3 (2.4)	1 (0.8)	7 (5.5)	6 (4.8)	1 (0.8)	0.233
12-60 (n=248)	30 (12.1)	27 (10.9)	13 (5.2)	20 (8.1)	12 (4.8)	8 (3.2)	0	14 (5.6)	11 (4.4)	3 (1.2)	0.269
>60 (n=146)	26 (17.8)	19 (13.0)	7 (4.8)	13 (8.9)	10 (6.8)	3 (2.1)	0	6 (4.1)	5 (3.4)	1 (0.7)	0.096
**LRTI**	62 (24.8)	39 (15.6)	23 (9.2)	22 (8.0)	15 (6.1)	7 (2.8)	3 (1.2)	18 (7.2)	12 (4.8)	6 (2.4)	0.509
**Total samples (n=250)**											
**Age (months)**											
≤12 (n=127)	22 (17.3)	12 (9.4)	10 (7.9)	9 (7.1)	5 (3.9)	4 (3.1)	2 (1.6)	4 (3.1)	2 (1.6)	2 (1.6)	0.154
12-60 (n=105)	33 (31.4)	22 (20.9)	11 (10.5)	9 (8.6)	6 (5.7)	3 (2.9)	1 (0.9)	13 (12.4)	9 (8.6)	4(3.8)	0.472
> 60 (n=2)	0 (0)	0 (0)	0 (0)	0 (0)	0 (0)	0 (0)	0 (0)	0 (0)	0 (0)	0 (0)	

^a^The incidence and percentage of RV genotypes A, B, and C in the population are an underestimate, since the genotype of only 73 (80%) of the 92 RV-positive samples could be determined.

^b^The percentage in all cases are referred to the total number of samples.

^c^In bold are the statistically significant differences.

On the other hand, 250 hospitalized children with clinical diagnosis suggestive of viral pneumonia were included in the study. Nasal washings were obtained and tested for the presence of respiratory viruses. Sixty-two children (41 males and 21 females, age range 1 to 76 months) were positive for RV (24.8%). The species of 43 (69.4%) of these viruses was determined by sequencing the 5'-UTR region of the viral genome. Of the typed viruses, 22 (51.2%) were RV-A, 18 (41.8%) RV-C, and 3 (7%) were RV-B (Table 1). The difference in the incidence of RV-A and RV-C was not statistically significant. RV-positive children were detected more frequently (p < 0.0001) in children between 25 and 36 months as compared to the other age groups (Table 1). There was no significant gender between children infected with RV or another respiratory virus or between RV-A and RV-C. Hospitalization for RV was significantly higher (p < 0.0001) in the spring as compared to other seasons.

### Co-infection of RV with other respiratory viruses

An additional respiratory virus was found in 30% (28/92) and 37% (23/62) of RV-positive patients with URTI and LRTI, respectively (Table 1). The virus species could be determined in 19 (14 RV-A, 5 RV-C) and 14 (7 RV-A, 1 RV-B, 6 RV-C) of URTI and LRTI mixed infections, respectively. Neither RV-A nor RV-C types were significantly associated with a particular respiratory virus in mixed infections of URTI or LRTI. However, in URTI, RV-A was found more frequently in co-infections than RV-C (p = 0.025). In both children populations, when RV was present in mixed infections, it was more frequently found associated with a single virus (URTI, 20/28; LRTI, 21/23) than with 2 other viruses (URTI, 8/28; LRTI, 2/23) (URTI p = 0.014, LRTI p < 0.0001).

### Clinical characteristics of RV-positive children

The major symptoms and signs observed in children with RV-associated URTI in single or mixed infections were cough, rhinorrhea, and sore throat. However, rhinorrhea was the only symptom significantly higher in RV-positive children as compared to patients with infections caused by other respiratory viruses (Table 2). Increased respiratory rate was found in more than one third of the patients with a single or mixed infection with RV, as well as in infections caused by other viruses. The clinical features of children having a co-infection with RV and other respiratory virus were no statistically different from those with single RV infections. Also, no significant difference was found between patients infected with RV species A or C. RV-B was excluded from the analysis given that a single child was infected with this virus.

**Table 2:** **Clinical observations in RV-positive children with URTI**

	**Other virus (%) (n = 284)**	**RV-positive (%) (n = 92)**	***p-value*** ^***a***^	**RV single infection (%) (n = 64)**	***p-value*** ^***a***^
Asthma^b^	50 (17.6)	19 (20.7)	0.512	14 (21.9)	0.426
Allergic rhinitis^b^	68 (23.9)	29 (31.5)	0.149	22 (34.4)	0.085
Rhinorrhea	232 (81.7)	83 (90.2)	**0.05**	57 (89.1)	0.156
Dysphagia	187 (65.8)	50 (55.3)	**0.047**	34 (53.1)	**0.049**
Cough	253 (89.1)	84 (91.3)	0.544	58 (90.6)	0.718
Nausea	66 (23.2)	13 (14.1)	0.062	10 (15.6)	0.183
Vomiting	74 (26.1)	12 (13.3)	**0.01**	7 (10.9)	**0.01**
Diarrhea	29 (10.2)	7 (7.6)	0.461	4 (6.3)	0.328
Headache	114 (41.5)	20 (21.7)	**0.001**	14 (21.9)	**0.006**
Myalgia/Arthralgia	85 (29.9)	11 (12.0)	**0.001**	9 (14.1)	**0.01**
Dysphonia	40 (14.1)	14 (15.2)	0.788	11 (17.2)	0.526
Nasal flaring	11 (3.9)	4 (4.3)	0.84	4 (6.3)	0.398
Intercostal retraction	36 (12.7)	10 (10.9)	0.646	9 (14.1)	0.765
Conjunctivitis	24 (8.5)	8 (8.7)	0.942	5 (7.8)	0.867
Xiphoid retraction	10 (2.5)	3 (3.3)	0.905	3 (4.7)	0.657
Thoracoabdominal dissociation	11 (3.9)	2 (2.2)	0.438	2 (3.1)	0.776
Dyspnea	38 (13.4)	11 (12.0)	0.724	10 (15.6)	0.638
Wheezing	53 (18.7)	14 (15.2)	0.453	12 (18.8)	0.987
Fever > =38	146 (51.4)	28 (29.3)	**0.0001**	17 (26.6)	**0.0001**
Increased heart rate^c^	25 (8.8)	3 (3.7)	0.078	2 (3.1)	0.125
Increased respiratory rate - tachypnea^c^	148 (52.1)	39 (42.4)	0.105	28 (43.8)	0.227

^a^p-values are between overall RV infections or RV in single infection and the presence of other respiratory virus. In bold are statistically significant differences.

^b^Previous condition.

^c^Adjusted by age range.

In 92.7% of the RV-positive patients with LRTI the diagnosis of pneumonia was confirmed radiologically, as it was in 94.5% of children positive for other viruses (Table 3). Children with RV had fever >38°C and increased respiratory rate more frequently than children with any other respiratory virus. This was true for single and mixed RV infections. Other major signs detected upon examination of RV-positive patients were thoracoabdominal dissociation, and intercostal retraction. According to the Silverman-Anderson score there was no difference between children with RV or any other virus; 76.4% of the patients with RV co-infections and 82.3% of RV single infections had a score between 1 and 3. In the chest-X ray examination, 78.1% of the RV-positive patients had signs of interstitial pneumonia; these percentages were similar for RV-single infections. There was no difference between the clinical signs observed in children infected with either RV-A or RV-C.

**Table 3:** **Clinical observations in RV-positive children with LRTI**

	**Other virus (%) (n = 126)**	**RV-positive (%) (n = 55)**	***p-value*** ^***a***^	**RV single infection (%) (n = 34)**	***p-value*** ^***a***^
Pneumonia	120 (94.5)	51 (92.7)	0.488	30 (88.32	0.131
Cough	121 (96.0)	50 (90.9)	0.165	32 (94.1)	0.628
Nasal flaring	65 (51.6)	19 (34.5)	**0.034**	10 (29.4)	**0.047**
Grunting on exhalation	29 (23.0)	8 (14.5)	0.194	6 (17.6)	0.502
Intercostal retraction	111 (88.1)	45 (81.8)	0.260	28 (82.4)	0.378
Xiphoid retraction	68 (54.0)	14 (25.5)	**0.0001**	10 (29.4)	**0.011**
Thoracoabdominal dissociation	51 (40.5)	24 (43.6)	0.691	12 (35.3)	0.583
Fever > =38	37 (29.4)	25 (45.5)	**0.036**	17 (50.1)	**0.024**
Increased respiratory rate - tachypnea^b^	94 (75.2)	49 (89.1)	**0.037**	32 (94.1)	**0.0001**

^a^p-values are between overall RV infections or RV in single infection and the presence of other respiratory virus. In bold are statistically significant differences.

^b^Adjusted by age range.

### Genetic diversity of RV

**Figure 2:**
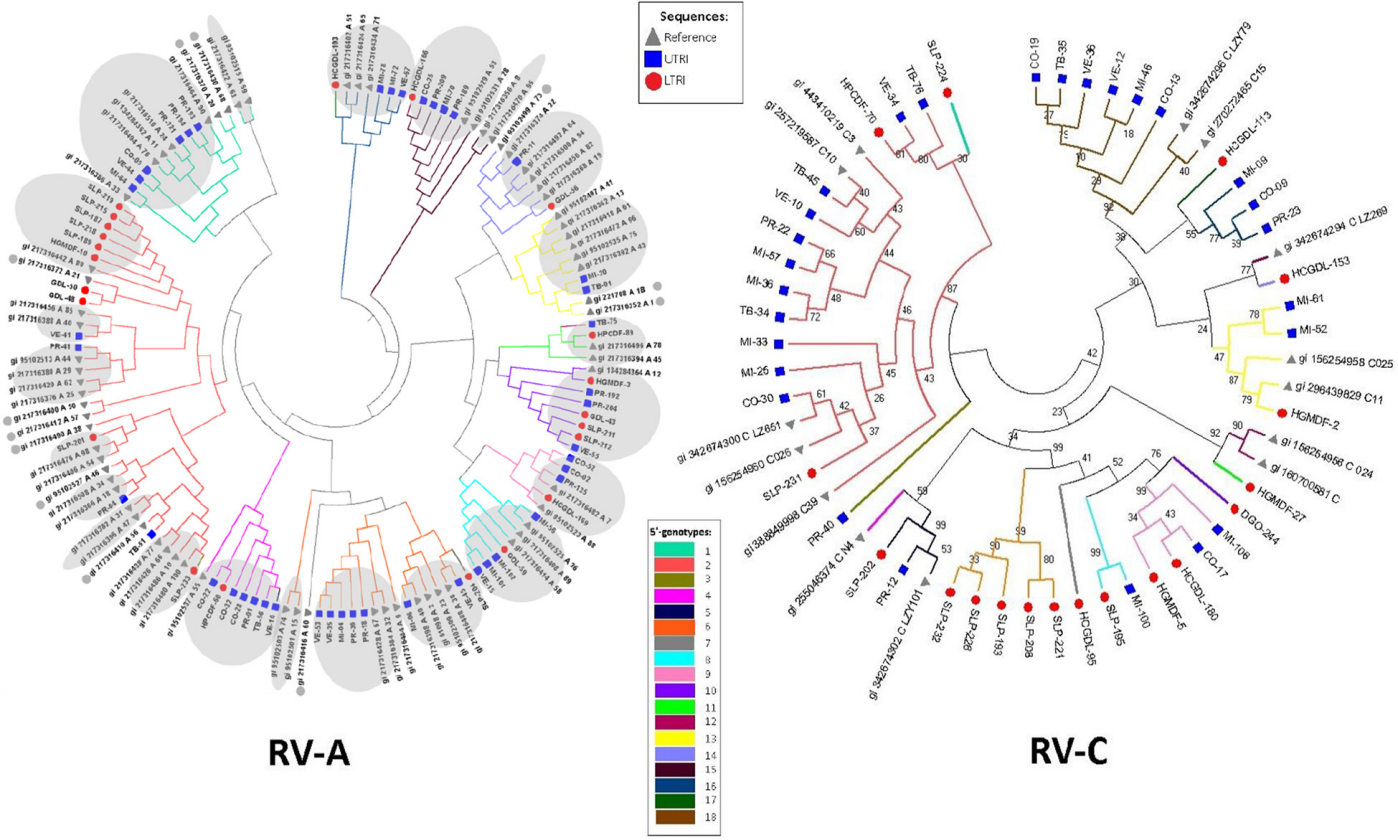
**Phylogenetic trees of clinical viral isolates based on the analysis of 400 nt from the 5′-UTR hypervariable region of the RV genome.** The tree branches of each 5′-genotype in the RV-A and RV-C trees are labeled with a different color; genotype numbering starts at the gap in the circle and increases counterclockwise. The names of the sequences starting with “gi” correspond to reference strains downloaded from GenBank and are depicted with grey triangles. The blue squares represent viruses detected in outpatient children, and their names start with the two initial letters of the city where the sample was collected (CO, Córdoba; MI, Minatitlán; PR, Poza Rica; TB, Tierra Blanca; VE, Veracruz). The red circles indicate viruses detected in hospitalized children, and the names of the hospitals from which the sample was collected are coded as follows: DGO, Hospital General de Durango; HCGDL, Hospital Civil de Guadalajara; HGMDF, Hospital General de México, D.F.; HPCDF, Hospital Pediátrico de Coyoacán, D.F.; GDL, Hospital de Pediatría, IMSS; SLP, Hospital Central de San Luis Potosí. The gray circles in the phylogenetic tree for RV-A species represent the terminal clades of the tree that were identical to the terminal clades of the tree constructed with full genomic sequences [6]. The reference strains (gi) that are not contained in the gray circles are marked with a dot at the end of the name, and represent sequences that do not match with the terminal clades of the reference, full genomic sequences tree. The database of reference strains contained only those viruses with complete genomic sequences. The numbers after the colors in the vertical bar represent the 5′-genotype of the virus, as described in the -Genetic diversity of RV- section of results.

The phylogenetic trees in Figure 2 depict the wide distribution of 5′-UTR sequences of the RV-A and RV-C strains isolated from URTI and LRTI patients. The 112 characterized RV strains could be classified into 35 different 5′-UTR-based genotypes (5′-genotypes); in this work, two 5′-genotypes were defined as different when their 5′-UTR sequence identity was equal or less than 87%, similar to the cutoff determined for genotypes based on VP1 nucleotide sequences [21]. Seventeen genotypes were associated with RV-A and 18 with RV-C. Each genotype included from 1 to 14 different virus strains. In the case of RV-A, 5′-genotypes were significantly more frequently found in either hospitalized (genotype 2, p < 0001) or outpatient (genotype 6, p < 0.001) children, while for RV-C some 5′-genotypes also associated more frequently with either URTIs (genotypes 2, p < 0.001 and 18, p = 0.03) or LRTIs (genotype 6, p < 0.01) (Figure 2). Importantly, the correlation of 5′-genotypes with reference genotypes defined by full genomic sequences [6] was remarkably high. Comparison of the phylogenetic trees constructed with either the 5′-genotypes or the full genomic sequences showed that 83% (12/72) of the terminal clades present in the 5′-phylogenetic tree were the same as compared with the terminal clades of the reference tree (Figure 2). These results suggest that 5′-genotypes could serve as a simpler and initial approach to genomically classify RVs. Further studies need to be conducted to determine if the association between some RV 5′-genotypes and the severity of infection observed in this work holds, or if it is the result of particular RV genomic types that circulated more frequently in the years that URTI and LRTI samples were collected.

## Discussion

Here, we report the frequency of RV in pediatric patients with URTI and LRTI in different regions of Mexico. The frequencies observed in this work are in agreement with those reported in other studies, in which RV has varied from 16% to 37% in children with either URTI or LRTI. Regarding seasonality, RV infections were associated most frequently to URTI in autumn and early spring, as reported [22], although it should de noted that the samples from patients with URTI miss the months from May to August. On the other hand, a clear peak of prevalence of the virus in LTRI was observed only during spring.

An additional respiratory virus was found in about one-third of the RV-positive patients with either URTI or LRTI. Detection of several viruses in a high proportion of cases has been a characteristic of respiratory infections in which a PCR-based diagnostic method was used. In particular, RV has been found in dual infections with another respiratory virus in 26% to 63% of cases [14,18,20]. However, the clinical relevance of detection of several viruses in pneumonia, and the association with severe illness is uncertain [2,23-25].

Despite technological advances, establishing the cause of pneumonia remains challenging [26]. In this study diagnosis of viral pneumonia relied on nasopharyngeal specimens, what might be misleading since detection of a virus in the nasopharynx could represent a coincidental upper-respiratory infection, the asymptomatic presence of the virus, or a genuine pneumonia pathogen. Measurement of background prevalence of asymptomatic nasopharyngeal viral infections in a healthy control group and in patients with mild-to-moderate disease might help to clarify the relevance of this diagnostic issue at a population level [2]. In most of this type of studies, but not in all, a higher RV detection among participants with illness was noted [11,13,19,27-32].

The overall identification of RV was similar but statistically different between the two patient populations studied. Children with URTI had a RV rate of 17% as compared to 24.5% in patients with LRTI (p < 0.017). The observation that the frequency of RV detection in serious respiratory disease is similar to that observed in mild-to-moderate illness, or even in asymptomatic cases [11,13,19,27-32], is intriguing. One would think that if the respiratory disease caused by RV was more severe than other pathogens, the percentage of hospitalized children positive for RV should be significantly higher than that found in outpatient or healthy children, as observed, for instance, in rotavirus infections [33]. On the contrary, the incidence of RV should have to be significantly lower in LRTI as compared to URTI if the virus infection caused a mild disease. Then, why is there about the same incidence of RV found in respiratory infections with different degrees of severity? It is tempting to hypothesize that specific virus serotypes/genotypes are preferentially associated to severe or mild clinical symptoms. This potential association might be obscured by a wide diversity of circulating RV serotypes/genotypes with potentially different intrinsic virulence, such that in different children populations similar frequencies of RV infection are observed but with different clinical outcomes. It cannot be discarded, however, that mixed infections with bacteria (not searched in this work) or different host factors could influence the clinical severity of RV infections, as reported [34]. Of interest, there have been suggestions since the earliest epidemiological studies of variation of virulence among the different RV serotypes [35,36] and, in this line, we noted in this work that some 5′-genotypes were detected only in either hospitalized or outpatient children. It would be important to characterize the diversity of RV genotypes in different parts of the world to determine if the prevalent viruses vary among regions and in time, and associate with different severity of the disease. Identification of this potential association would have a great impact in the development of prophylactic measures to control the infection of this very common pathogen.

In conclusion, this study underlines the high RV exposition and diversity of circulating strains in Mexico and the potential severity of disease associated with both RV-A and RV-C infections.

## Conclusions

This study describes the frequency of detection of rhinovirus species in children with upper and lower respiratory tract infections in Mexico and their genetic diversity, determined by sequencing the 5′ UTR region of the viral genome. We report that the 5′UTR sequence can be useful for an initial approach to determine the rhinovirus genotypes. We also describe the clinical characteristics of illness associated with specific RV species. We found that both RV-A and RV-C species were very frequently found in both hospitalized and outpatient children and no statistically significant differences were found in the severity of disease associated with RV-A and RV-C infections in neither of the two children populations. This study underlines the high RV prevalence and genetic diversity of circulating strains in Mexico and the potential severity of disease associated with both RV-A and RV-C infections. This is of particular relevance, since the information about respiratory viruses in Mexico is very limited, and studies characterizing viruses circulating in the community level are even scarcer.

## Methods

### Study populations and clinical samples

Two patient populations were included in this study. The first was composed of pediatric patients that attended the private consult in five different cities of the state of Veracruz, Mexico (Poza Rica, Veracruz, Córdoba, Tierra Blanca, and Minatitlán). The children were enrolled in the study if they were younger than 16 years, clinically diagnosed as having an acute respiratory infection, with an onset of illness less than one week, and the parent or guardian signed the informed consent form. Since none of these children required hospitalization, they were all considered as having URTIs. From September 2011 to April 2012 nasopharyngeal swabs (rayon-tipped, BD BBL) were collected from 526 children (male:female ratio, 1.27; median age, 19 months; average age, 39 months; age range, 0–191 months). The second population consisted of children that required hospital admission with suspected pneumonia in four cities (Guadalajara, Mexico City, San Luis Potosí, and Durango) in different states of Mexico. These children were all considered to have LRTIs. Nasal washings were collected from 250 children (male:female ratio, 1.43; median age, 10 months; average age, 16 months; age range, 1–76 months, with only one child 143 months old) between March 2010 and April 2011. For both populations, demographic and clinical information was collected. The samples were placed in vials containing viral transport medium (Microtest M4-RT, Remel, Lenexa, KS) and sent either to the Institute of Biotechnology in Cuernavaca (URTI samples) or to the School of Medicine in Mexico City (LRTI samples) and stored at **−**70°C until analyzed. The study was approved by the institutional review boards of the School of Medicine and the Institute of Biotechnology of the National University of Mexico and from the institutional review board and ethics committee of each participant hospital. Written informed consent was obtained from each parent or guardian prior to enrollment.

### Nucleic acid extraction and RT-PCR assay

Nucleic acids were extracted from 100 μl of respiratory specimens using the PureLink Viral RNA/DNA mini kit (Invitrogen, Carlsbad, CA). RV and other fourteen respiratory viruses were detected with the Seeplex®RV15 OneStep ACE Detection kit (Seegene, Seoul, South Korea) following the manufacturer’s instructions. This diagnostic test detects the following viruses: parainfluenza viruses 1, 2, 3, and 4, adenovirus A/B/C/D/E, human coronaviruses 229E/NL63 and HuCoV-OC43, rinovirus A/B/C, influenza A, influenza B, respiratory syncyctial virus-A and -B, human bocavirus 1/2/3/4, human metapneumovirus, and enterovirus. The RNA present in the samples positive for RV was reverse transcribed with random hexamers using standard protocols. PCR was then performed with previously described primers DK001 and DK004, which target a fragment of approximately 400 bp of the hypervariable region of the RV 5′-UTR virus genome [37]. The amplified DNA fragment was purified with the High Pure PCR Product Purification kit (Roche).

### Sequence analysis

The purified PCR products were sequenced in an Applied Biosystems (Foster City, CA), model 3130xl apparatus. The sequences obtained were edited using the Unipro UGENE and Seaview softwares. A database of RV complete genomes was created using all sequences available on GenBank until September 2013. BLAST was performed and the genotype of the RV strains was assigned based on the first five best scores. The 5′ sequences of the RV strains characterized in this work were deposited in GenBank with accession numbers KJ765008 to KJ765123.

### Phylogenetic analysis

Multiple sequence alignments were made using MUSCLE method; maximum likelihood trees were generated with 1000 repetitions bootstrap using the MEGA 5.2.2 program. To determine the 5′-genotypes, clustering was made using the cd-hit-test at 87% of identity.

### Statistical analysis

All statistical data analyses were carried out using PASW statistical software version 18.0 (SPSS Inc.). Significant differences between groups were evaluated using chi-square tests when it was possible, or Fisher’s exact tests when values were smaller than 25. Associations of demographic and clinical features of children with a) RV detected versus other respiratory virus and, b) RV-A versus RV-C, were examined using logistic regression. To determine the 5′-genotypes that were significantly enriched for RV-A or RV-C, the probability of randomly selecting different viral strains in each 5′-genotype was calculated by a combinatorial approach. In all statistical analysis p-values <0.05 were considered statistically significant.

## Consent

Written informed consent was obtained from each parent or guardian prior to enrollment in the study.
